# Supplementary material for: Praziquantel and risk of visual disorders: Case series assessment
Source: PLoS Negl Trop Dis. 2020 Apr 15;14(4):e0008198. doi: 10.1371/journal.pntd.0008198 (PMC7185715; doi:10.1371/journal.pntd.0008198)
Supplement: Attachment — Submitted filename: Comment20200103.docx [file pntd.0008198.s001.docx]

Comments

The manuscript “Praziquanel and Risk of Visual Disorders: Case Series Assessment” presents 61 case reports of visual abnormalities that arise within the first 24 hours of praziquantel administration. This adverse side effect is rare, and even the evidence of causal association between praziquantel and visual disorders is not enough, this paper still can give an alarm to the health personnel of other countries, especially the countries continuing mass chemotherapy for schistosomiasis and other parasitic diseases.

To get the sufficient evidence for confirming that praziquantel has this side effect, some trials should be done, especially double-blind studies. In the early of the 1980s, I did a double-blind study on praziquantel therapeutic effect and side effects. In the praziquantel group, the rate of side effects was 46.4 % and in the placebo group was 43.5%, and there was no significant difference between the two groups. So I suggest, in discussion, authors may add such meaning if the condition is allowed.

In addition, the English of the paper still need to be modified. For example “in the time period between January 2017 to August 2019” should be “in the time period between January 2017 and August 2019”. (Line 85).

Thanks
